# The landscape of management and outcome of children with congenitally corrected transposition of the great arteries: a single-center experience

**DOI:** 10.3389/fcvm.2025.1682795

**Published:** 2025-12-08

**Authors:** Yazeed Saleh Alahmed, Abdulraouf M. Z. Jijeh, Abdulsalam M. Alsaiad, T. Mesud Yelbuz, Mohamed S. Kabbani, Abdullah A. Alghamdi, Fahad Alhabshan, Ibrahim J. Alibrahim

**Affiliations:** 1 Department of Cardiac Sciences, Ministry of the National Guard—Health Affairs, Riyadh, Saudi Arabia; 2 King Abdullah International Medical Research Center, Riyadh, Saudi Arabia; 3 King Saud bin Abdulaziz University for Health Sciences, Riyadh, Saudi Arabia; 4 Department of Pediatrics, College of Medicine, Qassim University, Qassim, Saudi Arabia; 5 Department of Cardiology, Qassim University, Medical City, Qassim, Saudi Arabia

**Keywords:** congenitally corrected transposition of the great arteries (ccTGA), single-ventricle palliation, pulmonary artery banding, one-and-a-half ventricle, Rastelli operation

## Abstract

**Objective:**

To compare the outcomes in patients with congenitally corrected transposition of the great arteries (ccTGA) who were offered different surgical management strategies.

**Design and settings:**

We retrospectively included all patients from our center diagnosed with ccTGA between 2000 and 2021. The cohort was divided into four groups: (1) patients with systemic right ventricle; (2) patients with anatomic repair (in the form of an atrial and arterial switch or an atrial switch and Rastelli operation); (3) patients with a one-and-a-half ventricle repair (hemi-Mustard type) and bidirectional cavopulmonary connection and placement of a right ventricle to pulmonary artery conduit; and (4) patients with a single ventricle who were not suitable for biventricular repair.

**Results:**

The study included 42 patients with ccTGA. In 39 patients (93%), ccTGA was associated with other congenital heart defects. Group 1 (systemic right ventricle) comprised 16 patients (38%); seven of them underwent pulmonary artery banding, with a reduction in tricuspid valve regurgitation seen in four patients. Group 2 (anatomic group) and Group 3 (one-and-a-half ventricle group) each had three patients (7%). Group 4 (single-ventricle palliation) consisted of 17 patients (40%); 15 of them (88%) completed total cavopulmonary connection. Three early mortalities (7%) were recorded.

**Conclusions:**

Patients who underwent anatomical repair (Rastelli-type) or a one-and-a-half ventricle repair (hemi-Mustard type) showed a good outcome with satisfactory biventricular function. Moreover, single-ventricle palliation showed excellent mid-term results in patients with ccTGA. PA banding may reduce tricuspid regurgitation in patients with ccTGA. A follow-up of our managed patients by an individualized plan demonstrated satisfactory outcomes.

## Introduction

Congenitally corrected transposition of the great arteries (ccTGA) is an uncommon complex cardiac anomaly representing less than 1% of congenital heart malformations (1). It is characterized anatomically by the presence of atrioventricular (AV) and ventriculoarterial (VA) discordance. The morphological left ventricle (mLV) supports the pulmonary circulation, while the morphological right ventricle (mRV) supports systemic circulation (1).

The clinical presentation of patients with ccTGA is highly variable; the clinical picture is based on the presence of associated lesions. These lesions exist in over 90% of patients with ccTGA. The most frequent associated lesions include ventricular septal defect (VSD), left ventricular outflow tract (LVOT) obstruction, pulmonary stenosis (PS), anatomic and functional tricuspid valve (TV) abnormalities, and congenital heart block (2–4).

Current management strategies have two main principles, consisting of either physiologic or anatomic repair. However, the ideal approach for patients with ccTGA remains unclear (4–7). Physiologic “classic” repair focuses on repair of associated defects, leaving the mRV serving as the systemic ventricle, while AV and VA discordances are preserved. Although classic physiologic repair may result in a good short-term outcome, there are still some concerns with this strategy, mainly related to progressive tricuspid regurgitation (TR) and dysfunction of the right ventricle (RV) in the long term (5, 7–10).

The anatomic repair approach aims to restore blood flow through a normal sequence of anatomic structures based on correcting AV and VA discordances, plus associated cardiac defects (atrial switch and an arterial switch procedure (double-switch operation). A subgroup of patients whose anatomy may not be suitable for biventricular repair might undergo the so-called single-ventricle (SV) palliation pathway (11, 12). The success of the double-switch operation depends on the ability of the mLV to support systemic circulation. In the absence of an unrestrictive VSD and pulmonary arterial hypertension (PAH), the mLV will be deconditioned and may become unable to undertake the workload of systemic circulation (11–13). In patients with ccTGA without PS, the anatomic repair involves a double-switch operation, along with repair of any VSD present, while in patients with subpulmonary ventricular outflow tract obstruction, anatomic repair will be achieved by Rastelli-type surgery, which includes an atrial switch, VSD repair with tunneling of the left ventricle (LV) to the aorta, and placement of a RV to pulmonary artery (PA) conduit (RV-PA conduit) (2, 11, 12).

A meta-analysis conducted by Alghamdi et al. reported that Rastelli-type repair was associated with lower in-hospital mortality and lower incidence of postoperative heart block compared with arterial switch operation and physiologic repair (2).

When the mRV serves as a systemic ventricle, it may ultimately fail as the patient grows. The primary manifestation of its failure is progressive TR; therefore, pulmonary artery banding has been used as a transitory measure to stabilize TR and to train the LV before anatomic repair in the future. It has also been used in patients with VSD to minimize the excessive pulmonary blood flow that may potentially lead to PAH (6–13).

There are limited data from our region discussing the management of ccTGA.

Our study aimed to report our data and compare the outcomes in patients with ccTGA who were offered different surgical management strategies, including the SV palliation pathway.

## Methods

We included all patients in our center who were diagnosed with ccTGA between 2000 and 2021. Patients with right or left isomerism, tricuspid or mitral valve atresia, or isolated ventricular inversion were excluded.

Retrospectively, we reviewed demographic and clinical data, including the echocardiography reports, operative reports, and clinical records of all patients. The cohort was divided into four groups: (1) patients with a systemic right RV who were followed up only medically, including patients who underwent a Blalock–Thomas–Taussig (BTT) shunt or PA banding without further surgical intervention; (2) patients with anatomic repair in the form of an atrial and arterial switch operation (double-switch) or atrial switch and Rastelli operation; (3) patients with a one-and-a-half ventricle repair (hemi-Mustard type) and bidirectional cavopulmonary connection (BCPC) and placement of an RV to PA conduit; and (4) patients with SV who were not suitable for biventricular repair.

Reintervention was defined as the need for catheter-based or surgical intervention after definitive repair. Perioperative mortality was defined as death before hospital discharge or within 30 days after surgical or catheter-based intervention. Echocardiographic evaluations at the first study (before the first intervention) and at the last follow-up were reviewed. The systemic ventricular function was graded qualitatively as normal, mild, moderate, or severely depressed. Systemic AV valve morphology and function were examined, and regurgitation was graded qualitatively as absent, mild, moderate, or severe. Significant valvular regurgitation was defined when regurgitation was moderate or above moderate.

## Data management and analysis

The data were expressed as numbers and percentages for categorical variables and as mean ± standard deviation for continuous variables. Data that did not fit a normal distribution were expressed as median and interquartile range (IQR). A value of *P* < 0.05 was considered statistically significant. The statistical analysis was performed using SPSS for Windows, version 16.0 (SPSS Inc, Chicago, IL, USA).

## Results

A total of 42 patients with ccTGA who met our inclusion criteria were studied; out of these, 28 patients were male (67%) and 14 were female (33%). The median age at presentation was 2 months (IQR: 0.2–20 months). The median follow-up duration was 7 years (IQR: 4.2–11 years) (Table 1).

**Table 1:** Patients' demographics.

Parameter	Systemic RV (*n* = 16)	Anatomical or one-and-a-half ventricle repair (*n* = 6)	Single ventricle (*n* = 17)	*p*
Age at first surgery (months)	19 (6–87)	4 (0.6–27)	14 (2–36)	0.47
Age at second procedure (months)	26 (2.2–NA)	46 (12–68)	24 (9–41)	0.86
Weight at first surgery (kg)	11 (4–18)	5 (3–9)	9 (4–12)	0.3
Weight at second procedure (kg)	13 (9–NA)	10 (7–15)	11 (7–14)	0.87
Age at last follow-up (years)	8 (4–16)	10 (6–13)	6 (5–9)	0.25

NA, not available due to insufficient data to produce the limit.

Because of the small number of patients in Groups 2 and 3, the two groups were combined into one group (anatomical or one-and-a-half ventricle repair) for the purposes of demographic description.

Associated congenital heart defects in ccTGA are summarized in Table 2. Three patients had situs inversus (7%), two of them had pulmonary atresia, and one had severe valvular pulmonary stenosis. Dextrocardia was seen in seven patients (17%), out of which four were females, and mesocardia was seen in four patients (10%), out of which three were females. Five patients had mildly depressed (systemic) RV function at the time of initial echocardiogram. Three patients had isolated ccTGA, and all were followed up medically (systemic RV group). Two patients had complete heart block and required permanent pacemaker (PPM) insertion, one at the time of diagnosis and one at the age of 7.

**Table 2:** Associated congenital heart defects in ccTGA.

Variable	Number (Percentage)
Total cases of ccTGA, *n* (%)	42 (100%)
ccTGA with associated CHD	39 (93%)
Isolated ccTGA	3 (7%)
VSD	35 (83%)
ASD	27 (64%)
LVOT obstruction	25 (60%)
Mild to moderate PS	7/25 (28%)
Severe PS	13/25 (52%)
Pulmonary atresia	5/25 (20%)
Situs inversus	3 (7%)
TV regurgitation	33 (79%)
Ebsteinoid systemic TV^a^	13 (31%)
Mild TR	23 (70%)
Moderate TR	9 (27%)
Severe TR	1 (2.4%)
Mitral valve regurgitation^b^	6 (14%)
Coarctation of aorta^c^	6 (14%)
Dextrocardia	7 (17%)
Mesocardia	4 (10%)
Brady arrhythmia required PPM	12 (29%)
Complete heart block	10 (24%)
Sinus node dysfunction	2 (5%)
Left SVC	5 (12%)
Left SVC to CS	3 (7%)
Left SVC to LA	2 (5%)
RVOT obstruction (subaortic)	4 (10%)
Right aortic arch	4 (10%)
Straddling of mitral valve	4 (10%)
Single right coronary artery	3 (7%)

ASD, atrial septal defect; CHD, congenital heart disease; CS, coronary sinus; LA, left atrium; LVOT, left ventricular outflow obstruction; PS, pulmonary stenosis; RV, right ventricle; RVOT, right ventricular outflow obstruction; PPM, permanent pacemaker; SVC, superior vena cava; TV, tricuspid valve; VSD, ventricular septal defect.

a 50% of patients with Ebsteinoid TV had moderate to severe TR.

b Four out of six patients had mitral valve straddling.

c All of them had a left aortic arch.

**Figure 1:**
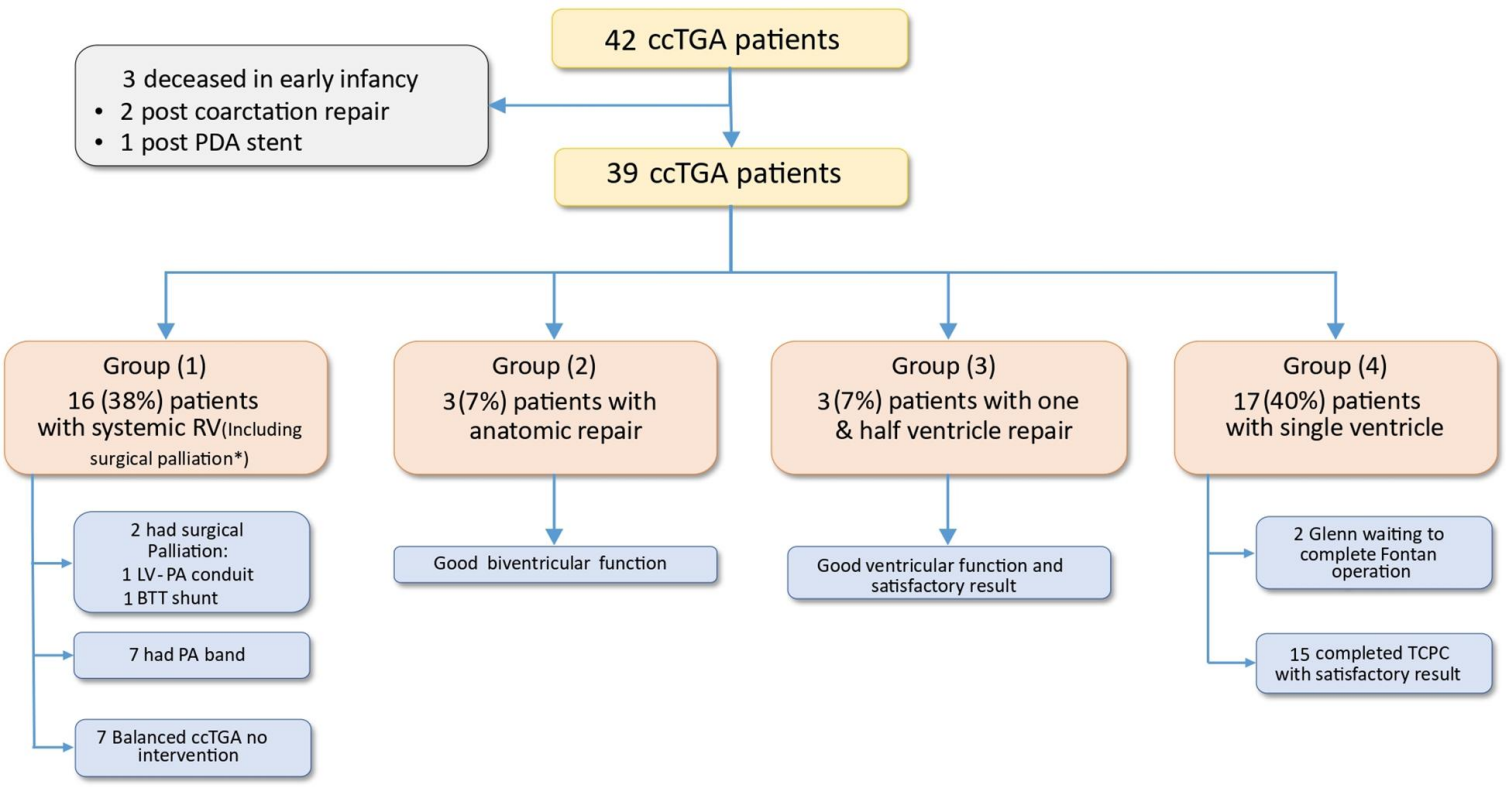
A flowchart demonstrating the overall management strategy of ccTGA. BTT shunt, Blalock–Thomas–Taussig shunt; ccTGA, congenitally corrected transposition of the great arteries; LV, left ventricle; PA, pulmonary artery; PDA, patent ductus arteriosus; RV, right ventricle; TCPC, total cavopulmonary connection. *Patients underwent a PA band, a BTT shunt, and an LV-PA conduit only (where the RV remains systemic).

**Figure 2:**
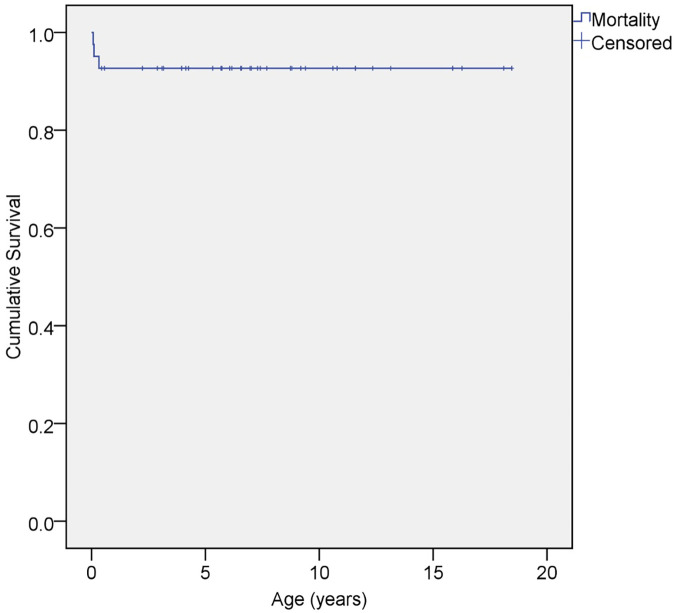
A Kaplan–Meier chart for the survival of ccTGA patients in our cohort.

Only one patient was lost to follow-up. Three newborns (7%) suffered early mortality. Two of them had a severe coarctation of the aorta with depressed ventricular function and required an aortic arch repair, but they did not survive postoperatively. The third patient had pulmonary atresia, for which a patent ductus arteriosus (PDA) stent was placed to augment the pulmonary blood flow. He was discharged in stable condition but succumbed 23 days later at home (Figures 1 and 2).

Group 1 (systemic RV group), which included patients who underwent a PA banding and a BTT shunt or an LV-PA conduit only (where the RV remains systemic), comprised 16 patients (38%). Seven patients had balanced ccTGA and did not require surgical intervention; all had mild to moderate TR at last follow-up. Seven patients had no LVOT obstruction and significant TR and underwent PA banding (median age of banding, 14.8 months; IQR, 5.5–23 months). The primary reason for carrying out PA banding was to train the LV for possible future anatomic repair. The severity of TR lessened in four of them (57%) after the PA banding; the median follow-up duration was 6 years (IQR: 4–11 years). One patient underwent LV to PA conduit at the age of 7 months because of original progressive LVOT obstruction, and one patient underwent a BTT shunt because of severe LVOT obstruction as palliative therapy. The systemic ventricle (RV) function was mildly to moderately depressed in 4 patients out of 16 (25%) (Figure 1).

Group 2 (anatomic repair group) included three patients (7%). Biventricular repair was achieved by the Senning operation, LV to the aorta tunnel, and RV to the PA conduit. All patients were naturally banded by the presence of moderate to severe LVOT obstruction. All patients had good overall biventricular systolic function and no significant tricuspid valve regurgitation during follow-up.

Group 3 (one-and-a-half ventricle group) included three patients (7%). One of them was diagnosed at the age of 2 with severe LVOT obstruction and underwent a BCPC “Glenn procedure” and then a total cavopulmonary connection (TCPC) “Fontan procedure” at the age of 3. However, because of a failing Fontan, this patient was redirected to a one-and-a-half ventricle repair in the form of Fontan takedown, a hemi-Mustard procedure (baffling of inferior vena cava (IVC) to TV), and an RV to PA conduit while keeping the Glenn anastomosis at 6 years of age. The other two patients underwent a hemi-Mustard procedure, a Glenn procedure, and an arterial switch operation after initial palliation with a PA band. All patients had good overall biventricular systolic function and no significant TR during follow-up.

Among six patients (Groups 2 and 3) who had RV-PA conduit “Centegra”, two required reoperation for RV to PA conduit replacement after an average of 8 years from the initial surgery, and the remaining four patients had good function RV-PA conduit with a median follow-up of 8 years (IQR: 3–11).

Group 4 (single-ventricle group) consisted of 17 patients (40%). Fifteen patients had completed the Fontan procedure (among them, three patients, who presented at 6, 9, and 17 years of age respectively, underwent primary TCPC as a one-stage procedure), and two were waiting for the Fontan procedure after the Glenn operation. One patient required a reoperation for systemic ventricle outflow tract obstruction at the age of 5. Two patients had a mildly depressed ventricular function, and none had significant TR.

## Discussion

Current strategies for managing ccTGA have two main principles: either physiologic repair (where the RV remains to serve as the systemic ventricle) or anatomic repair (a double-switch operation). Although the physiologic repair strategy may yield a favorable short-term outcome, concerns are associated with this approach, primarily related to the long-term progression of TR and RV dysfunction (4, 7–10). In 16 patients from our study, the RV served as the systemic ventricle with good RV systolic function in 75% of them and a mild to moderately depressed RV function in 25%. Similarly, De León et al. reported that ventricular systolic function remained stable in the majority of patients who underwent physiologic repair (6). Comparable results were reported in other literature as well (1, 5).

Reduction in TR was reported in patients who had PA banding placed to train the LV for longer than 6 months. The theory is that the increase in LV pressure caused by banding alters the ventricular septal geometry, pushing the septal leaflet of the TV into the RV, which leads to an improvement of TV coaptation and a decrease in RV preload (14–16). However, Winlaw et al. noted that while PA banding improved the ventricular systolic function, it did not reduce TR in patients before repair (17). Contrastingly, Brawn et al. reported that PA banding resulted in a deterioration of LV systolic function in trained groups of patients who underwent the double-switch operation (13).

In our patient population, TR reduced in more than 50% of those who underwent palliation by PA banding. Similar results were reported by De Léon et al., who described 22 patients who underwent PA banding; in 8 of them, TR reduced, while in 11, it remained unchanged. They also reported that in the remaining three patients, TR worsened. The ventricular systolic function remained stable in the majority of their patients (6). PA banding may be considered for longer-term palliation, which is supported by evidence from the literature. In a review of 40 patients comparing the outcomes of anatomical repair (double-switch) vs. long-term PA banding palliation, the latter resulted in lower mortality and better cardiac function as well as better clinical status (16). Similar findings were reported by Cools et al. (18) Some recent studies have identified PA banding before the performance of the double-switch operation as a risk factor for the development of aortic root dilation and aortic regurgitation after the operation, which might contribute to late LV dysfunction (13, 16, 19). In addition, Duncan reported that PA banding may cause myocardial necrosis and fibrosis in addition to ventricular systolic dysfunction (11).

Seventeen of our patients with ccTGA underwent a single-ventricle pathway; there was no mortality or significant TV dysfunction. Only two patients had a mildly depressed function, and one patient required a reoperation for LVOT obstruction. Hraska et al. reported a superior outcome of SV palliation in 17 patients who had a complex ccTGA anatomy (7). De Léon et al. reported better survival rates in patients with ccTGA who underwent the Fontan procedure during a median follow-up of 11 years. None of their patients required further reintervention, and they all remained with good ventricular systolic function at the last follow-up. Moreover, almost all of them had minimal AV valve regurgitation (6). We have observed similar results for our Fontan patients; however, long-term follow-up is required to adequately assess the outcomes of the SV palliation strategy for patients with ccTGA.

A recent retrospective multicenter study reported excellent long-term survival and low rates of atrioventricular valve failure and heart failure in individuals with ccTGA who underwent Fontan completion (155/194 patients) (20).

Out of the three mortality cases we reported, two patients had CoA, requiring neonatal repair. Kalfa et al. reported that CoA and hypoplasia of the aortic arch were significantly associated with mortality (20).

Ideally, the goal in patients with ccTGA is to establish a biventricular circulation in which the LV supports systemic circulation; however, this cardiac anomaly may present with a broad spectrum of associated lesions, which usually affects the surgical decision (2–4).

In our series, patients who underwent this pathway had ccTGA, VSD, and PS. Therefore, biventricular repair was achieved by a Rastelli-type operation, including tunneling of the LV to the aorta, the RV to the PA conduit, and atrial switch. Our patients who underwent anatomical repair or a one-and-a-half ventricular repair maintained a good outcome. Two out of six patients required reintervention for RV-PA conduit replacement at an average of 8 years. Similar results were reported in the literature (6, 7). Bogers et al. found that while Rastelli-type repair resulted in less postoperative heart block, this type of operation carried a higher risk for reoperation, mainly for RV-PA conduit replacement at a mean age of 9 years (21).

.Barrios et al. reported in their series of 240 patients that those with physiologic repair had comparable survival rates to those with anatomic repair at early to mid-term (12 years postrepair). Still, the curve will change direction as the overall survival rate was lower among patients who underwent physiologic repair (71% at 15 years) (22).

Our study has many limitations. It is a retrospective analysis limited by the available information, as it is a single-center study. The decision to proceed with each surgical pathway was dependent on many factors; thus, we attempted to address this by reviewing weekly surgical decision forms and operative notes for each patient. The median follow-up was 7 years (IQR: 4.2–11 years), and a longer follow-up duration is required to assess the single-ventricle palliation group.

## Conclusions

More than 90% of patients with ccTGA had associated congenital heart defects (complex ccTGA). The spectrum of the management of ccTGA may include PA banding as palliative surgery for patients with significant TR. In the small number of patients who underwent anatomical repair (Rastelli-type) or a one-and-a-half ventricle repair (hemi-Mustard type), outcomes were good at mid-term follow-up. Single-ventricle palliation showed excellent mid-term results in patients with ccTGA. A follow-up of our managed patients with an individualized plan demonstrated a satisfactory outcome.

## Data Availability

The original contributions presented in the study are included in the article/Supplementary Material, and further inquiries can be directed to the corresponding author.
